# Development and validation of a prognostic nutritional-inflammatory-immune score and web-based dynamic nomogram for gastric cancer following radical gastrectomy: a multicenter study

**DOI:** 10.3389/fnut.2026.1858192

**Published:** 2026-08-06

**Authors:** Changxin Yu, Zhen Wang, Shanshan Feng, Hongjuan Cao, Dandan Gong, Lixia Zhang, Wei Bo, Hao Guo, Xiaoyang Jiang, Xinghao Ma

**Affiliations:** 1 Department of Nutrition, School of Public Health, Anhui Medical University, Hefei, Anhui, China; 2 Department of Clinical Nutrition, Lu'an Hospital, Anhui Medical University, Lu’an, Anhui, China; 3 Department of Clinical Nutrition, Chuzhou Hospital, Anhui Medical University, Chuzhou, Anhui, China; 4 Department of Chronic Non-communicable Diseases Prevention and Control, Lu’an Municipal Center for Disease Control and Prevention, Lu’an, Anhui, China; 5 Department of Clinical Nutrition, Taihe Hospital, Wannan Medical College, Fuyang, Anhui, China; 6 Department of Clinical Nutrition, Yingshang County People's Hospital, Fuyang, Anhui, China; 7 Department of Clinical Nutrition, Anqing Hospital, Anhui Medical University, Anqing, Anhui, China

**Keywords:** dynamic nomogram, gastric cancer, immunity, inflammation, nutrition

## Abstract

**Introduction:**

Although nutritional, inflammatory, and immune status are key factors in gastric cancer (GC) prognosis, tools integrating these three dimensions for survival prediction remain limited. We developed and internally validated a prognostic nutritional-inflammatory-immune (PNII) score and a dynamic nomogram to predict overall survival (OS) after radical gastrectomy.

**Methods:**

A total of 841 patients from five centers who underwent radical gastrectomy were randomly divided into a training cohort (*n* = 588) and an internal validation cohort (*n* = 253). The PNII score was constructed from 12 candidate preoperative biomarkers using LASSO Cox regression. A prognostic nomogram integrating the PNII score with clinicopathological variables was developed and internally validated.

**Results:**

The PNII score, comprising nutritional, inflammatory, and immune markers, was identified as an independent prognostic factor. High PNII scores were associated with adverse tumor characteristics and poorer OS. The nomogram incorporating PNII, age, pT stage, pN stage, and CA125 achieved a C-index of 0.754 in both cohorts and outperformed the base clinical model without PNII (ΔAUC = 0.014; DeLong *p* = 0.0325). Time-dependent ROC analysis yielded 5-year AUCs of 0.831 (training) and 0.823 (validation), and decision curve analysis indicated net clinical benefit.

**Conclusion:**

The PNII-based dynamic nomogram may serve as a promising postoperative risk-stratification tool for patients with GC. Independent external validation and prospective clinical evaluation are required before routine clinical use.

## Introduction

Gastric cancer (GC) continues to pose a major public health burden, ranking fifth among the most prevalent malignancies globally and imposing a substantial strain on healthcare systems (1). Radical gastrectomy remains the primary curative treatment for resectable GC (2, 3). However, long-term survival still varies substantially even after complete resection, largely due to differences in individual physiological status and variations in treatment response (4). Consequently, an accurate assessment of postoperative prognosis is crucial for informing adjuvant therapy decisions and tailoring postoperative surveillance.

Clinically, GC prognosis is primarily assessed using the 8th edition AJCC pTNM staging system. However, the pTNM staging system focuses primarily on tumor-related characteristics and fails to adequately incorporate the patient’s systemic status, including key clinicopathological variables and blood-based biomarkers (5). As a result, prognostic assessment based solely on pTNM staging may be suboptimal, increasing uncertainty in individualized treatment planning. Therefore, there is a need for prognostic tools that integrate systemic host status with tumor staging to inform personalized management and improve overall survival (OS) among GC patients.

Accumulating evidence indicates that the patient’s systemic nutritional, inflammatory, and immune status is an important determinant of long-term prognosis in GC. Several nutritional parameters, including the hemoglobin-albumin-lymphocyte-platelet (HALP) score, prognostic nutritional index (PNI), and neutrophil-albumin ratio (NAR), demonstrate reliable prognostic value. Similarly, inflammatory and immune biomarkers, including the systemic immune-inflammation index (SII), lymphocyte-to-monocyte ratio (LMR), platelet-to-lymphocyte ratio (PLR), and neutrophil-to-lymphocyte ratio (NLR), are strongly associated with patient survival (6–11). Nevertheless, the prognostic performance of any single biomarker remains limited. This limitation underscores the need to integrate these variables into a more comprehensive prognostic model to optimize risk stratification.

To address this clinical need, we developed a prognostic nutritional-inflammatory-immune (PNII) score that integrates markers from these three domains to predict OS after radical gastrectomy for GC. To determine whether the PNII score provides additional prognostic information beyond individual indices, we evaluated its prognostic performance. We then constructed and internally validated a PNII-based dynamic nomogram integrated with clinicopathological variables for postoperative survival prediction.

## Materials and methods

### Study cohort

This research enrolled GC patients treated with radical gastrectomy from January 2019 to December 2022 across five institutions: Lu’an People’s Hospital, Anqing Municipal Hospital, Chuzhou First People’s Hospital, Taihe County People’s Hospital, and Yingshang County People’s Hospital. The primary endpoint was OS, defined as the time from surgery to death from any cause; surviving patients were censored at last follow-up. The final data cutoff date was July 2025. Eligible patients met the following criteria: (1) age ≥18 years; (2) GC confirmed by endoscopic biopsy; (3) receipt of radical gastrectomy; and (4) pTNM stage I-III disease. The exclusion criteria were: (1) R1 or R2 resection; (2) remnant gastric cancer; (3) palliative surgery; (4) missing critical clinicopathological data; and (5) receipt of neoadjuvant chemoradiotherapy. Ultimately, 841 eligible patients were randomly divided into training (*n* = 588) and validation (*n* = 253) cohorts at a 7:3 ratio (Supplementary Figure S1).

### Clinical data acquisition and variable definitions

We systematically extracted clinicopathological and perioperative data. Collected baseline characteristics consisted of age, sex, body mass index (BMI), lifestyle habits (smoking, drinking), and the Nutritional Risk Screening 2002 (NRS2002) score. Perioperative and surgical details comprised the American Society of Anesthesiologists (ASA) grade, operative time, surgical approach, resection extent, reconstruction type, intraoperative blood loss, perioperative blood and albumin transfusions, and combined organ resection. Pathological features incorporated tumor location and diameter, histologic differentiation, pTNM stage, pT stage, pN stage, as well as the presence of perineural or lymphovascular invasion.

Preoperative laboratory variables included hematological indices, such as neutrophil, monocyte, lymphocyte, red blood cell, platelet counts, and hemoglobin levels, as well as biochemical markers, including fibrinogen, albumin, globulin, total cholesterol, and fasting blood glucose. Tumor biomarkers assessed included AFP (0–8.78 ng/mL), CA125 (0–35 U/mL), and CA19-9 (0–37 U/mL). CEA cutoff values were defined according to smoking status (smokers: <10 ng/mL; non-smokers: <5 ng/mL). All cutoff values were based on the reference ranges adopted by each participating institution’s clinical laboratory.

Based on these parameters, 12 nutritional, inflammatory, and immune biomarkers were calculated: NLR, ALBI grade, SII, hemoglobin-to-red cell distribution width ratio (HRR), SIRI, PLR, PNI, NAR, HALP score, LMR, SIS, and FIB. The specific calculation formulas for these biomarkers are detailed in Supplementary Tables S1, S2. All hematological and biochemical data were obtained from peripheral blood samples taken within 1 week before the operation.

### PNII score construction

Individual laboratory parameters (e.g., hemoglobin, platelet count, albumin, lymphocyte count, neutrophil count) were not entered directly into the variable screening process. Instead, they were integrated into 12 composite hematological and inflammatory indices (e.g., HALP, HRR, PLR, NLR) that reflect nutritional, inflammatory, and immune status simultaneously. These composite indices were then screened as candidate predictors.

All continuous variables were standardized to z-scores (mean = 0, SD = 1) before entering the LASSO Cox regression, so that the L1 penalty applied without scale bias. Univariable Cox regression was first employed to screen nutritional, inflammatory, and immune biomarkers, retaining significant variables (*p* < 0.05). To minimize overfitting and select optimal predictors, LASSO Cox regression was applied to these candidate variables. Multicollinearity among the selected features was evaluated using the variance inflation factor (VIF) to ensure statistical independence. The PNII formula was built from these standardized inputs; each coefficient therefore reflects the variable’s penalized contribution in the multivariate model rather than its unadjusted univariate association with mortality.

### Prognostic value of the PNII score

To assess the association between the PNII score and clinicopathological characteristics, score distributions were compared among different patient subgroups using the Wilcoxon rank-sum or Kruskal-Wallis test, as appropriate. Time-dependent ROC curve analysis was performed to evaluate the PNII score’s prognostic value for 1-, 3-, and 5-year OS, yielding AUCs in both cohorts. The optimal PNII score cutoff was identified via X-tile software, classifying patients into high- and low-risk groups. Univariable and multivariable Cox regression models were applied to identify independent prognostic factors.

### Construction and validation of a nomogram

Before multivariable Cox regression, the generalized variance inflation factor (GVIF) was calculated to assess multicollinearity among candidate predictors. Multivariable Cox regression analysis was subsequently performed to identify independent prognostic factors. A dynamic nomogram predicting OS was constructed in the training cohort by incorporating these independent prognostic factors. Model performance was assessed across both cohorts via calibration curves, time-dependent ROC curves, and the C-index. Decision curve analysis (DCA) assessed the clinical utility of the model relative to traditional pTNM staging.

The nomogram is intended for postoperative survival prediction, applicable once pathological staging is available after surgery. Users enter the five variables, and the calculator returns individualized 1-, 3-, and 5-year OS probabilities. The calculator is hosted on GitHub Pages and is freely accessible.

### Statistical analysis

Five variables had missing values: AFP (*n* = 72, 8.6%), BMI (*n* = 54, 6.4%), operative time (*n* = 24, 2.9%), intraoperative blood loss (*n* = 23, 2.7%), and tumor diameter (*n* = 17, 2.0%). All other variables, including the primary outcome, were complete. Missing values were imputed by Multiple Imputation by Chained Equations (MICE) with predictive mean matching, generating five imputed datasets (*m* = 5). The imputation model included all predictor variables, baseline demographic and clinical characteristics, the Nelson-Aalen estimator of the cumulative hazard, and the event indicator (12). To prevent information leakage, the dataset was split into training and validation cohorts before imputation, and imputation was performed independently within each cohort. Estimates from Cox models were pooled using Rubin’s rules.

**Figure 1:**
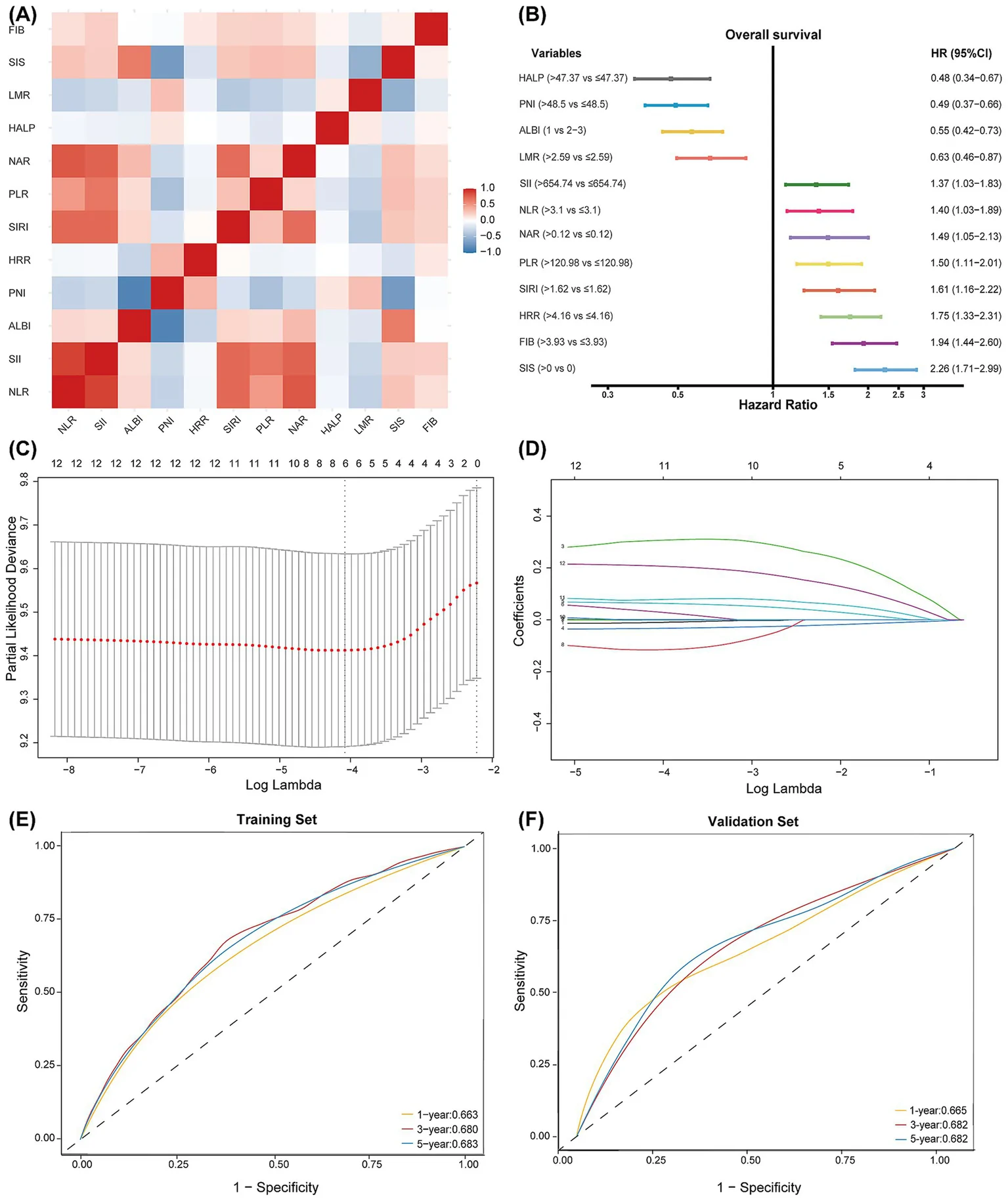
**(A)** Heatmap showing correlations among nutritional-inflammatory-immune related biomarkers. **(B)** Forest plot of univariable Cox regression analyses for OS. **(C)** Ten-fold cross-validation for tuning parameter selection in the LASSO Cox model; the red dots indicate the mean cross-validated partial likelihood deviance, the gray bars represent ±1 standard error (SE), and the vertical dotted line indicates the optimal value using the 1-SE criterion. **(D)** LASSO coefficient profiles of 12 candidate biomarkers. Time-dependent ROC curves for predicting 1-, 3-, and 5-year OS in the training set **(E)** and the validation set **(F)**. ROC: Receiver operating characteristic. All continuous variables were standardized (z-score) before formula calculation. Higher composite PNII scores consistently indicate greater mortality risk.

The normality of continuous variables was assessed with the Shapiro–Wilk test. Because the distribution of candidate biomarkers deviated from normality, Spearman’s rank correlation was used for all pairwise comparisons (Figure 1A).

X-tile software (Yale University, New Haven, CT, USA) identified 66 years as the optimal age cutoff using maximally selected rank statistics (Chi-square = 13.096, Miller-Siegmund corrected *p* = 0.0091). Patients were stratified into ≤66 and >66 years groups for all subsequent analyses.

Continuous baseline variables were analyzed using Student’s t-test or the Wilcoxon test, whereas categorical data were assessed with Chi-square or Fisher’s exact tests. Survival distributions were estimated via the Kaplan–Meier method with log-rank comparisons. All statistical computations were conducted in R software (version 4.5.2) and SPSS (version 27.0). Statistical significance was defined as a two-sided *p* < 0.05.

## Results

### Baseline characteristics and PNII score

Among the 841 patients, 601 (71.5%) were male and 240 (28.5%) were female, with a median age of 68 years (IQR: 62–74). The median follow-up duration reached 50 months (range: 1–90). During the follow-up period, 299 deaths were recorded (training: 207; validation: 92). For all 841 patients, the estimated OS rates at the 1-, 3-, and 5-year marks reached 88.2, 71.4, and 63.5%, respectively. The baseline characteristics of both cohorts demonstrated comparable distributions across all measured parameters (all *p* > 0.05; Table 1). Complete data were available for the primary outcome. Five variables had missing values (all <10%; Supplementary Table S3), which were handled via MICE as described in the Methods.

**Table 1:** Baseline clinicopathological characteristics of patients with gastric cancer after radical gastrectomy.

Variables	All patients (*N* = 841)	Training cohort (*N* = 588)	Validation cohort (*N* = 253)	*p* value
Age (years), median [IQR]	68 [62–74]	68 [62–73]	68 [61–74]	0.746
Sex				0.167
Male	601 (71.5%)	429 (73.0%)	172 (68.0%)	
Female	240 (28.5%)	159 (27.0%)	81 (32.0%)	
Smoking				0.860
No	670 (79.7%)	467 (79.4%)	203 (80.2%)	
Yes	171 (20.3%)	121 (20.6%)	50 (19.8%)	
Drinking				0.172
No	704 (83.7%)	485 (82.5%)	219 (86.6%)	
Yes	137 (16.3%)	103 (17.5%)	34 (13.4%)	
BMI (kg/m^2^)				0.320
<18.5	106 (12.6%)	79 (13.4%)	27 (10.7%)	
≥18.5	735 (87.4%)	509 (86.6%)	226 (89.3%)	
NRS2002				0.998
≥3	384 (45.7%)	269 (45.7%)	115 (45.5%)	
<3	457 (54.3%)	319 (54.3%)	138 (54.5%)	
Location				0.443
Lower third	244 (29.0%)	179 (30.5%)	65 (25.7%)	
Middle third	211 (25.1%)	140 (23.8%)	71 (28.1%)	
Mixed type	61 (7.3%)	42 (7.1%)	19 (7.5%)	
Upper third	325 (38.6%)	227 (38.6%)	98 (38.7%)	
ASA				0.934
>2	206 (24.5%)	145 (24.7%)	61 (24.1%)	
≤2	635 (75.5%)	443 (75.3%)	192 (75.9%)	
Operative time (min)	252 [210–300]	255 [215–296]	248 [210–300]	0.994
Surgical approach				0.987
Laparoscopic gastrectomy	371 (44.1%)	260 (44.2%)	111 (43.9%)	
Open gastrectomy	470 (55.9%)	328 (55.8%)	142 (56.1%)	
Resection extent				0.671
Subtotal gastrectomy	360 (42.8%)	255 (43.4%)	105 (41.5%)	
Total gastrectomy	481 (57.2%)	333 (56.6%)	148 (58.5%)	
Reconstruction type				0.387
Billroth I	52 (6.2%)	35 (6.0%)	17 (6.7%)	
Billroth II	90 (10.7%)	66 (11.2%)	24 (9.5%)	
Others	60 (7.1%)	47 (8.0%)	13 (5.1%)	
Roux-en-Y	639 (76.0%)	440 (74.8%)	199 (78.7%)	
Intraoperative blood loss (mL)	150 [100–200]	150 [100–200]	150 [100–200]	0.687
Blood transfusion				0.295
No	588 (69.9%)	418 (71.1%)	170 (67.2%)	
Yes	253 (30.1%)	170 (28.9%)	83 (32.8%)	
Albumin transfusion				0.295
No	242 (28.8%)	176 (29.9%)	66 (26.1%)	
Yes	599 (71.2%)	412 (70.1%)	187 (73.9%)	
Tumor diameter (cm)				0.858
>3.5	461 (54.8%)	324 (55.1%)	137 (54.2%)	
≤3.5	380 (45.2%)	264 (44.9%)	116 (45.8%)	
Histologic differentiation				0.464
Poorly differentiated	548 (65.2%)	378 (64.3%)	170 (67.2%)	
Well to moderately differentiated	293 (34.8%)	210 (35.7%)	83 (32.8%)	
pTNM				0.781
Stage I	252 (30.0%)	179 (30.4%)	73 (28.9%)	
Stage II	189 (22.5%)	134 (22.8%)	55 (21.7%)	
Stage III	400 (47.5%)	275 (46.8%)	125 (49.4%)	
pT				0.679
T1	181 (21.5%)	125 (21.3%)	56 (22.1%)	
T2	122 (14.5%)	90 (15.3%)	32 (12.7%)	
T3	184 (21.9%)	124 (21.1%)	60 (23.7%)	
T4	354 (42.1%)	249 (42.3%)	105 (41.5%)	
pN				0.096
N0	356 (42.4%)	254 (43.2%)	102 (40.3%)	
N1	149 (17.7%)	102 (17.4%)	47 (18.6%)	
N2	155 (18.4%)	97 (16.5%)	58 (22.9%)	
N3	181 (21.5%)	135 (22.9%)	46 (18.2%)	
Combined resection				0.756
No	744 (88.5%)	522 (88.8%)	222 (87.8%)	
Yes	97 (11.5%)	66 (11.2%)	31 (12.2%)	
Perineural invasion				0.418
No	521 (61.9%)	370 (62.9%)	151 (59.7%)	
Yes	320 (38.1%)	218 (37.1%)	102 (40.3%)	
Lymphovascular invasion				0.318
No	396 (47.1%)	284 (48.3%)	112 (44.3%)	
Yes	445 (52.9%)	304 (51.7%)	141 (55.7%)	
AFP (ng/mL)				0.827
High	46 (5.5%)	31 (5.3%)	15 (5.9%)	
Low	795 (94.5%)	557 (94.7%)	238 (94.1%)	
CEA (ng/mL)				0.356
High	171 (20.3%)	125 (21.3%)	46 (18.2%)	
Low	670 (79.7%)	463 (78.7%)	207 (81.8%)	
CA125 (U/mL)				1.000
High	51 (6.1%)	36 (6.1%)	15 (5.9%)	
Low	790 (93.9%)	552 (93.9%)	238 (94.1%)	
CA199 (U/mL)				0.843
High	111 (13.2%)	79 (13.4%)	32 (12.6%)	
Low	730 (86.8%)	509 (86.6%)	221 (87.4%)	
ALBI				0.449
1	556 (66.1%)	394 (67.0%)	162 (64.0%)	
2–3	285 (33.9%)	194 (33.0%)	91 (36.0%)	
SIS				0.437
0–1	563 (66.9%)	399 (67.9%)	164 (64.8%)	
2	278 (33.1%)	189 (32.1%)	89 (35.2%)	
NLR, median [IQR]	2.18 [1.60–3.11]	2.17 [1.61–3.09]	2.24 [1.60–3.18]	0.746
SII, median [IQR]	456 [304–710]	460 [306–700]	447 [296–760]	0.953
PNI, median [IQR]	48.30 [43.6, 52.3]	48.30 [43.8, 52.3]	48.30 [43.4, 52.2]	0.798
HRR, median [IQR]	3.43 [2.52, 8.89]	3.50 [2.58, 8.97]	3.17 [2.38, 8.62]	0.132
SIRI, median [IQR]	0.89 [0.56, 1.40]	0.88 [0.57, 1.34]	0.91 [0.54, 1.47]	0.615
PLR, median [IQR]	139 [104, 185]	139 [103, 189]	138 [104, 184]	0.845
NAR, median [IQR]	0.08 [0.06, 0.11]	0.08 [0.06, 0.11]	0.08 [0.06, 0.11]	0.485
HALP, median [IQR]	35.30 [22.0, 51.9]	34.60 [21.9, 52.4]	36.10 [22.0, 50.6]	0.944
LMR, median [IQR]	3.70 [2.78, 5.00]	3.71 [2.79, 5.00]	3.67 [2.77, 5.00]	0.800
FIB (g/L), median [IQR]	3.26 [2.70, 3.84]	3.28 [2.71, 3.85]	3.24 [2.70, 3.75]	0.435

BMI, body mass index; ASA, American Society of Anesthesiologists; ALBI, albumin-bilirubin grade; FIB, fibrinogen; HALP, hemoglobin-albumin-lymphocyte-platelet score; HRR, hemoglobin-to-red cell distribution width (RDW) ratio; LMR, lymphocyte-to-monocyte ratio; NAR, neutrophil-to-albumin ratio; NLR, neutrophil-to-lymphocyte ratio; PLR, platelet-to-lymphocyte ratio; PNI, prognostic nutritional index; SII, systemic immune-inflammation index; SIRI, systemic inflammation response index; SIS, systemic inflammation score.

Based on Kaplan–Meier survival curves, superior OS was significantly associated with a lower ALBI grade and lower levels of FIB, HRR, NAR, NLR, PLR, SII, SIRI, and SIS, as well as elevated HALP, LMR, and PNI (Supplementary Figure S2). Pairwise correlations among the 12 biomarkers are depicted in Figure 1A. Univariable Cox regression analysis demonstrated that each candidate biomarker exerted a significant prognostic impact on OS (*p* < 0.05; Figure 1B). LASSO Cox regression identified six biomarkers with non-zero coefficients (HRR, ALBI, FIB, SIS, PNI, and NAR) associated with postoperative prognosis (Figure 1C, 1D). Additionally, VIF analysis showed values <5 for all six variables, indicating the absence of significant multicollinearity (Supplementary Table S4). The PNII score was calculated according to the following equation:

$$\begin{array}{l} \text{Risk score}=\mathrm{HRR}\times 0.054+\text{ALBI}\times 0.222+\mathrm{FIB}\times 0.180\\ +\mathrm{SIS}\times 0.037-\mathrm{PNI}\times 0.035-\mathrm{NAR}\times 0.315 \end{array}$$

The coefficients in the PNII formula are penalized weights estimated in the multivariate standardized space. Because systemic inflammatory markers share substantial variance (e.g., NAR, SIS, FIB, and HRR), the sign of any single coefficient may not match its univariate prognostic direction. This sign reversal is a known consequence of regularized regression applied to correlated predictors and does not undermine the composite score: higher PNII scores consistently indicate higher predicted mortality risk across all cohorts.

We next examined the association between the PNII score and clinicopathological characteristics. Higher PNII scores were significantly associated with older age (≥66 years), larger tumor size (>3.5 cm), the presence of lymphovascular invasion, poor histological differentiation, and advanced pTNM stage. However, PNII scores did not differ significantly by sex (Supplementary Figure S3). These findings suggest that the PNII score reflects the overall physiological burden of GC patients.

**Figure 2:**
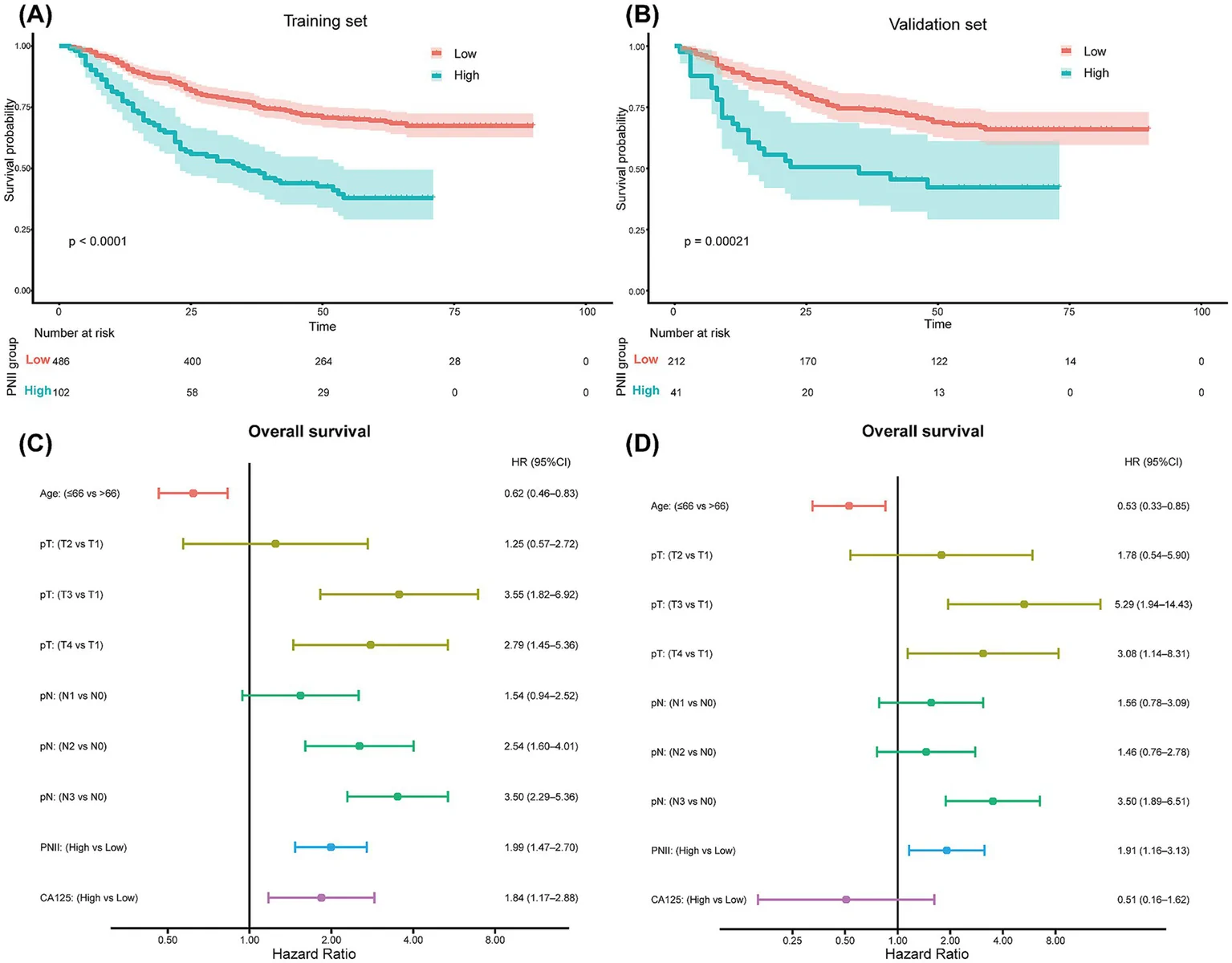
Prognostic value of the PNII score for OS in the training and validation cohorts. Kaplan–Meier curves of OS stratified by the PNII score are shown for the training cohort **(A)** and the validation cohort **(B)** with patients categorized into low- and high-risk groups according to the PNII score. **(C,D)** Forest plots of the multivariable Cox regression analyses for OS in the training cohort **(C)** and validation cohort **(D)**.

Time-dependent ROC analysis revealed stable discriminative accuracy of the PNII score across both cohorts, with 1-, 3-, and 5-year AUCs of 0.663, 0.680, and 0.683 (training; Figure 1E) and 0.665, 0.682, and 0.682 (validation; Figure 1F). In both cohorts, PNII showed higher AUCs than single blood-based biomarkers (Supplementary Figure S4). At an optimal cutoff of −1.09, patients were classified into low- and high-risk groups. Patients with high PNII scores exhibited significantly inferior OS across both cohorts based on Kaplan–Meier survival curves (*p* < 0.05; Figures 2A,B). To adjust for clinicopathological covariates, both univariable and multivariable Cox analyses were performed (Supplementary Table S5). PNII emerged as an independent prognostic factor for OS in multivariable analysis (training: HR 1.99, 95% CI, 1.47–2.70, Figure 2C; validation: HR 1.91, 95% CI, 1.16–3.13, Figure 2D).

### Construction and validation of the PNII nomogram

**Figure 3:**
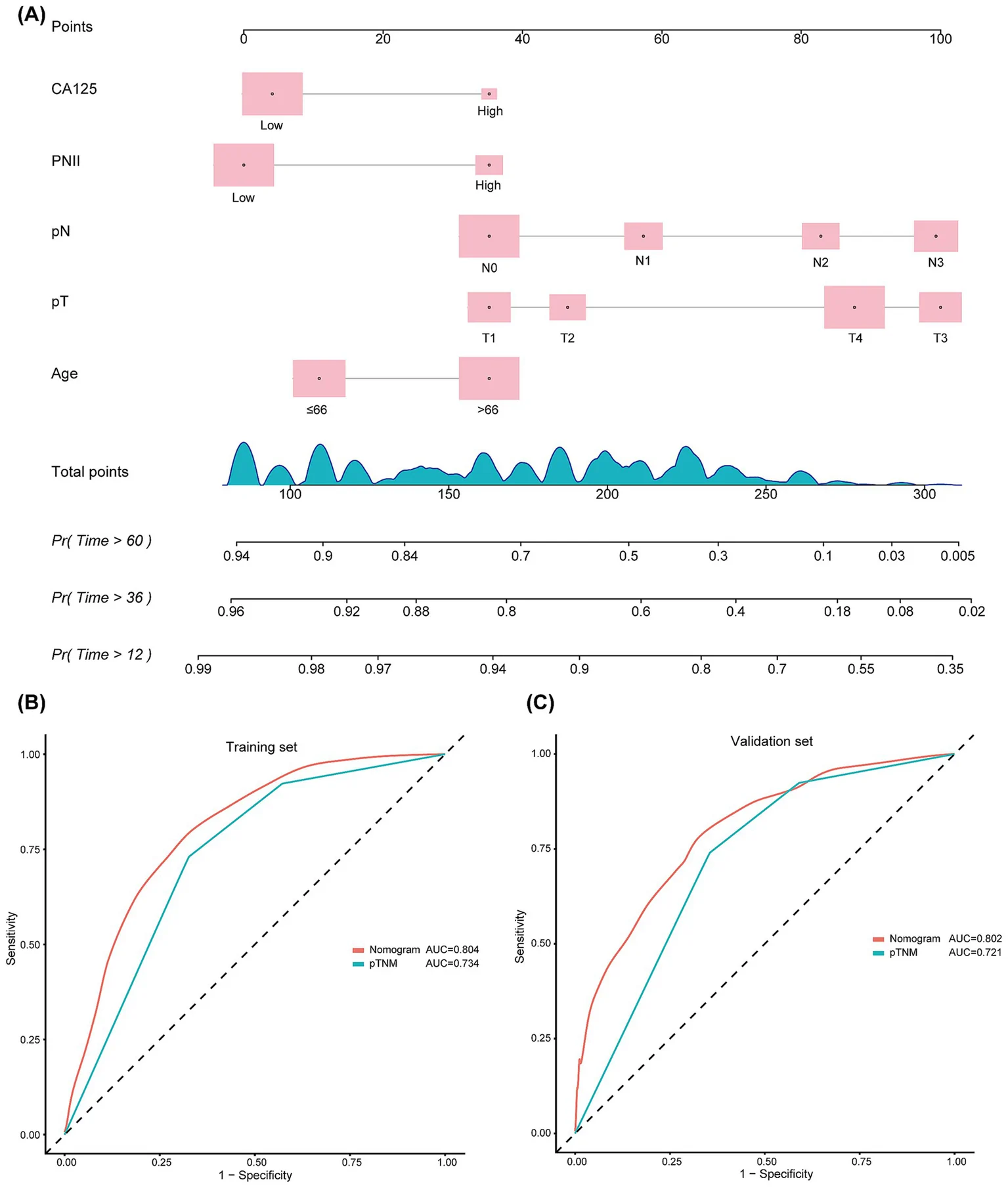
Construction and the validation of the nomograms. Nomograms incorporating the PNII score and other clinicopathological parameters for OS prediction in the training cohort **(A)**. Comparison of ROC curves between the nomogram and the pTNM staging system in the **(B)** training cohort and validation cohort **(C)**.

Multicollinearity was assessed using the GVIF, and all variables showed adjusted GVIF values <5, indicating no substantial multicollinearity (Supplementary Table S6). Consequently, we constructed a nomogram (Figure 3A) incorporating the PNII score, age, pT stage, pN stage, and CA125 for OS prediction in GC patients. The event distribution by CA125 status in both cohorts is summarized in Supplementary Table S7. To facilitate clinical application, we developed a web-based dynamic nomogram: https://yu861868-lab.github.io/PNII-nomogram-calculator/.

To isolate the contribution of the PNII score, we first compared the full nomogram (Age + pT + pN + CA125 + PNII) with the base clinical model that excluded only the PNII score (Age + pT + pN + CA125). The full model achieved a higher AUC (0.804 vs. 0.790; ΔAUC = 0.014; DeLong *p* = 0.0325), indicating that adding PNII improved discrimination.

DeLong comparisons against each individual variable further showed that the full nomogram outperformed pN stage (AUC = 0.738, *p* = 6.47 × 10^−6^), pT stage (AUC = 0.691, *p* = 3.17 × 10^−10^), PNII alone (AUC = 0.594, *p* = 2.37 × 10^−24^), age (AUC = 0.579, *p* = 2.27 × 10^−21^), and CA125 (AUC = 0.535, *p* = 1.09 × 10^−41^) (Supplementary Figure S5).

The nomogram achieved a C-index of 0.754 in both cohorts, compared with 0.693 and 0.668 for pTNM staging alone in the training and validation cohorts, respectively. The nomogram showed higher AUCs than the conventional pTNM system across the training (0.804 vs. 0.734) and validation (0.802 vs. 0.721) cohorts (Figures 3B,C). Time-dependent ROC analysis yielded consistent results: the nomogram AUCs at 1, 3, and 5 years were 0.741, 0.807, and 0.831 in the training cohort and 0.778, 0.801, and 0.823 in the validation cohort, respectively (Supplementary Figure S6), exceeding those of the pTNM staging system. Moreover, the nomogram improved estimation of OS compared with pTNM staging alone, with a continuous NRI of 0.477 (95% CI: 0.313–0.640; *p* < 0.001) and an IDI of 0.083 (95% CI: 0.057–0.108; *p* < 0.001).

**Figure 4:**
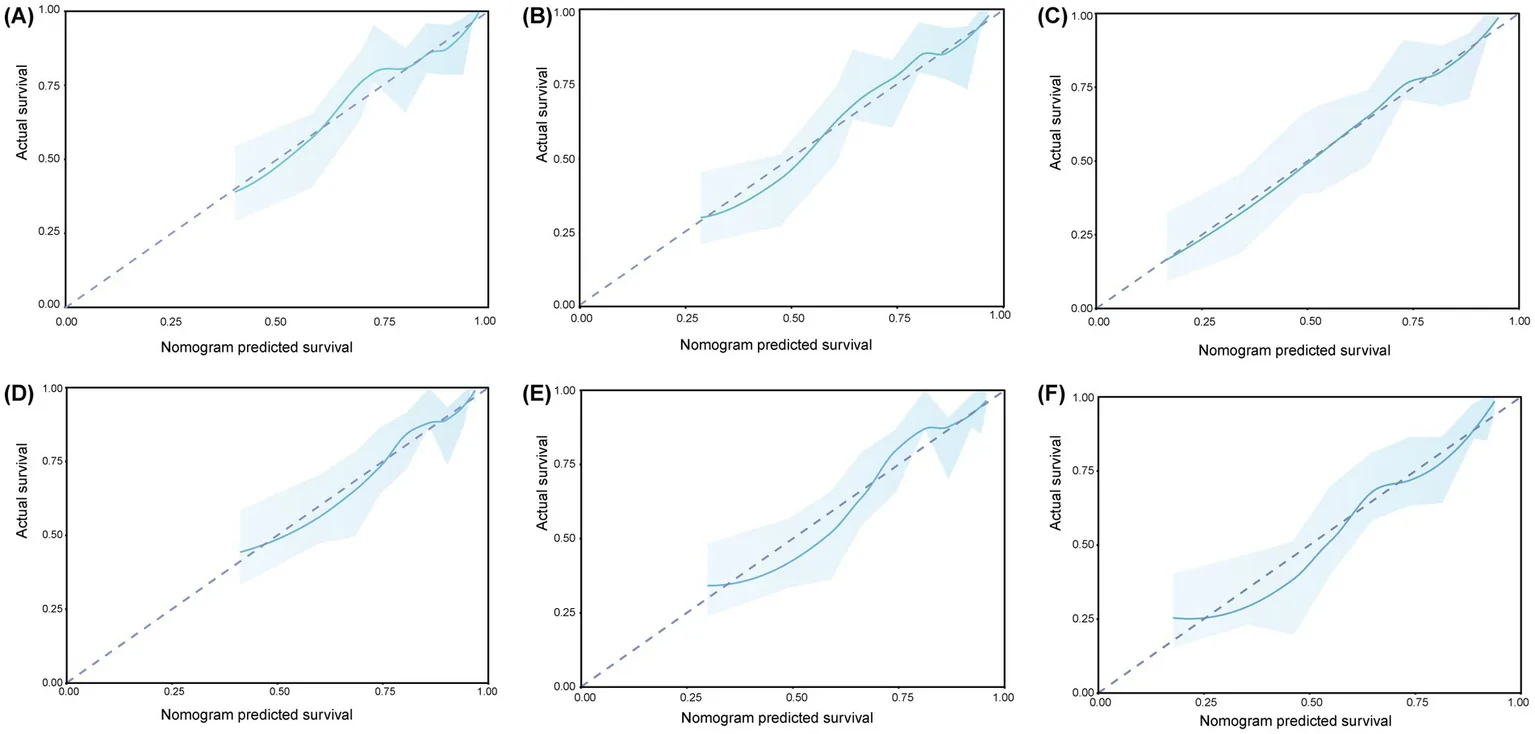
Calibration plots of the nomogram for predicting 1-, 3-, and 5-year OS. Training cohort **(A–C)**; Validation cohort **(D–F)**.

**Figure 5:**
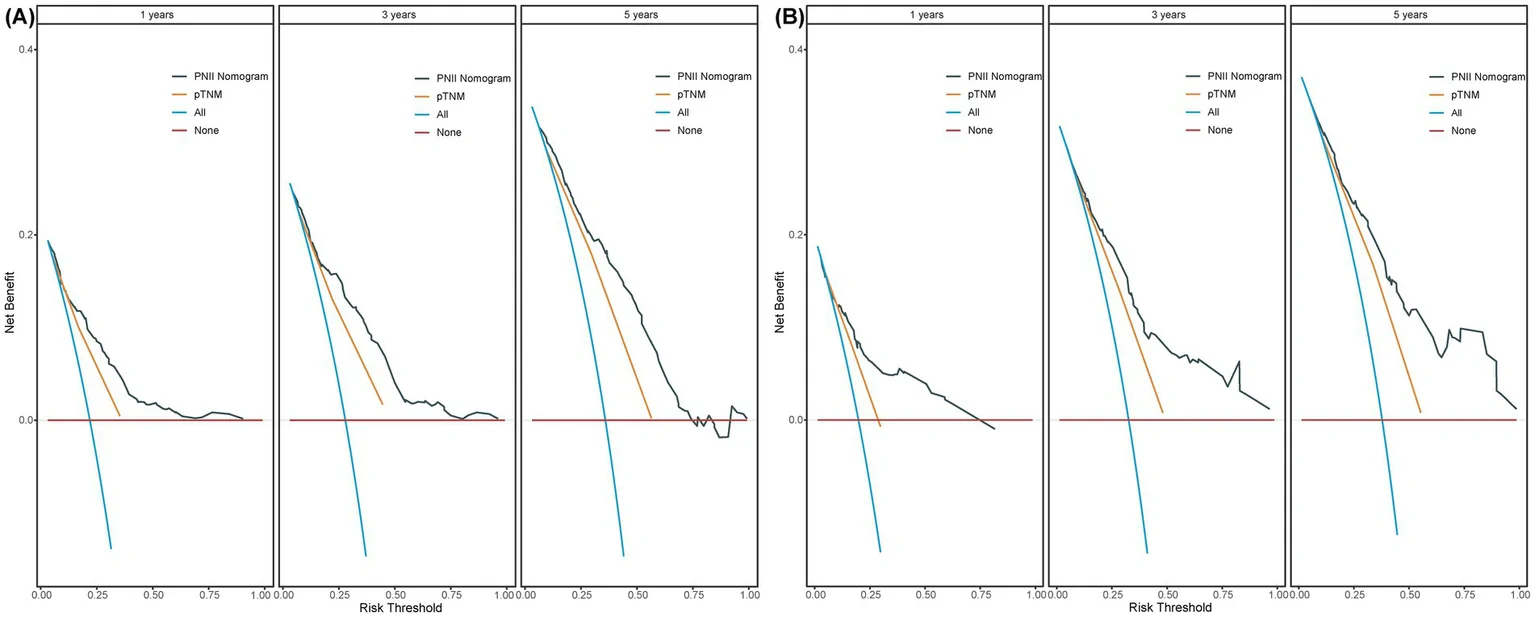
Decision curve analysis for predicting 1-, 3-, and 5-year OS. Training cohort **(A)**. Validation cohort **(B)**. The nomogram is compared with pTNM stage, treat-all, and treat-none strategies.

Calibration plots showed excellent agreement between predicted and observed OS (Figure 4). DCA demonstrated net benefit for the nomogram compared with pTNM staging across diverse threshold probabilities (Figure 5).

## Discussion

Although pTNM staging remains the cornerstone for prognostic evaluation, it does not account for patients’ nutritional, inflammatory, and immune status (13–15). Survival therefore varies considerably among patients with the same pTNM stage (16, 17).

To our knowledge, this is among the first studies to propose a composite score integrating nutritional, inflammatory, and immune biomarkers for prognostic prediction in GC. The PNII score was developed to integrate information from these three dimensions into a single prognostic metric. This composite score comprises six components: SIS, FIB, HRR, PNI, NAR, and ALBI. PNI reflects nutritional reserve and immune competence (6, 8, 18–20); SIS and FIB capture inflammatory and coagulation status (21–24); HRR integrates hemoglobin and red cell distribution width; NAR reflects the neutrophil-to-albumin balance; and ALBI grade stratifies hepatic functional reserve (25, 26). By combining these markers, the PNII score captures systemic host status more comprehensively than any single biomarker.

We therefore constructed and internally validated a nomogram incorporating clinical characteristics, preoperative hematological indices, and postoperative pathological features.

The nomogram incorporates patient age, two preoperatively assessable biomarkers (the PNII score and serum CA125 level), and two pathological features (pT stage and pN stage). Age was identified as an independent prognostic factor. The 66-year threshold was derived from this cohort. Other GC studies have reported different optimal age cutoffs, and the specific value depends on the population examined (27, 28). Older patients in this cohort may have had greater comorbidity burden and poorer functional reserve, which could contribute to higher non-cancer mortality (29–31).

CA125 was retained in the nomogram because it captures prognostic information distinct from the PNII score. While the PNII score reflects the host’s systemic condition, elevated CA125 is strongly associated with peritoneal dissemination, serosal invasion, and a high tumor burden — all of which portend a poor prognosis. Therefore, CA125 complements the model by adding a crucial tumor-burden dimension (32).

Beyond these preoperative indicators, the nomogram incorporates pT stage and pN stage. pT stage reflects depth of tumor invasion, with 5-year survival declining from 89.3% in pT1 to 23.7% in pT4 (33–35). Similarly, the extent of lymph node metastasis is a well-established prognostic factor, with 5-year survival declining from 94.9 to 41.8% as the positive node count increases from 0 to >6 (36–38). A preoperative-only model comprising age, PNII score, and CA125 showed modest discrimination (5-year AUC = 0.660), confirming that pathological staging contributes substantially to the nomogram’s performance and supporting its intended postoperative application.

NRS2002, a widely used nutritional screening tool, did not emerge as an independent prognostic factor in our multivariable analysis. Its items—weight loss and reduced oral intake—describe clinically apparent nutritional decline. The PNII score, by contrast, draws on serological biomarkers that reflect subclinical inflammation, immune competence, and coagulation status. These disturbances often develop before overt weight loss or reduced intake becomes evident, and they can shape tumor progression and postoperative recovery in ways that phenotypic screening tools cannot capture. The PNII score therefore adds prognostic information beyond what NRS2002 and similar tools provide.

In internal validation, the nomogram showed higher discrimination than the pTNM staging system, with a higher C-index and time-dependent AUC. NRI and IDI analyses indicated that the PNII score improved risk reclassification. Calibration showed good agreement between predicted and observed survival, and DCA suggested net clinical benefit.

This study has several strengths. The PNII score integrates nutritional, inflammatory, and immune markers into a single metric and is calculated from routinely available preoperative blood tests, making it feasible for clinical adoption without additional testing burden. The nomogram was developed using LASSO Cox regression to minimize overfitting, and the resulting web-based dynamic calculator facilitates bedside use. The study included a relatively large sample (*n* = 841) drawn from five centers, and internal validation was performed in a multicenter cohort.

Several limitations should be noted. First, the validation was based on random data partitioning rather than true external validation. Center-level partitioning was not pursued because the five centers differed substantially in sample size and case mix. Prospective studies with independent external cohorts are needed to test generalizability. Second, CA72-4 and several other established tumor biomarkers were excluded because missing data exceeded 50%, above our prespecified 20% threshold. Third, the number of patients with elevated CA125 was small (*n* = 51, 6.1%), and the stability of this variable should be confirmed in larger cohorts. Fourth, adjuvant chemotherapy, postoperative complications, recurrence, and comorbidity data were not systematically collected, representing an important source of residual confounding; the nomogram should therefore be interpreted as an associative rather than a causal prognostic tool. Fifth, BMI was dichotomized at 18.5 kg/m^2^ and may not fully capture prognostic information from higher BMI categories or nonlinear associations with survival. Finally, the retrospective design, the exclusively Chinese study population, and the absence of molecular features in the model remain limitations that future studies should address.

## Conclusion

The PNII score, derived from six routinely available preoperative biomarkers (HRR, ALBI, FIB, SIS, PNI, and NAR), integrates nutritional, inflammatory, and immune status into a single prognostic metric for patients undergoing radical gastrectomy for GC. In this multicenter cohort, the PNII score was independently associated with OS. A dynamic nomogram incorporating the PNII score with age, pT stage, pN stage, and CA125 demonstrated favorable discrimination and outperformed the base clinical model without PNII. The PNII-based dynamic nomogram may serve as a promising postoperative risk-stratification tool for patients with GC.

## Data Availability

The raw data supporting the conclusions of this article will be made available by the authors, without undue reservation.
