# Supplementary material for: Development and validation of a prognostic nutritional-inflammatory-immune score and web-based dynamic nomogram for gastric cancer following radical gastrectomy: a multicenter study
Source: Front Nutr. 2026 Aug 6;13:1858192. doi: 10.3389/fnut.2026.1858192 (PMC13489887; doi:10.3389/fnut.2026.1858192)
Supplement: Supplementary file 1 [file Data_Sheet_1.docx]

Supplementary Material

**Supplementary Figure S1.** Flow diagram of patient selection for the study.

**Supplementary Figure S2.** Kaplan-Meier curves for overall survival (OS) stratified by (A) ALBI grades, (B) FIB, (C) HALP, (D) HRR, (E) LMR, (F) NAR, (G) NLR, (H) PLR, (I) PNI, (J) SII, (K) SIRI, (L) SIS. ALBI grade, albumin-bilirubin grade; FIB, fibrinogen; HALP, hemoglobin-albumin-lymphocyte-platelet score; HRR, hemoglobin-to-red cell distribution width ratio; LMR, lymphocyte-to-monocyte ratio; NAR, neutrophil-to-albumin ratio; NLR, neutrophil-to-lymphocyte ratio; PLR, platelet-to-lymphocyte ratio; PNI, prognostic nutritional index; SII, systemic immune-inflammation index; SIRI, systemic inflammation response index; SIS, systemic inflammation score.

**Supplementary Figure S3.** Distribution of PNII scores across clinicopathologic characteristics. Comparisons between two groups were performed using the Wilcoxon rank-sum test, and comparisons among three groups were performed using the Kruskal-Wallis test.

**Supplementary Figure S4.** ROC curves comparing the predictive performance of PNII and single blood-based biomarkers for OS.

**Supplementary Figure S5.** Receiver operating characteristic (ROC) curves comparing the full nomogram with the base clinical model and each individual prognostic factor. (A) Full nomogram (Age + pT + pN + CA125 + PNII) vs. base clinical model (Age + pT + pN + CA125). (B) Full nomogram vs. PNII alone. (C) Full nomogram vs. age. (D) Full nomogram vs. pT stage. (E) Full nomogram vs. pN stage. (F) Full nomogram vs. CA125. DeLong test P values are reported in each panel. AUC, area under the curve.

**Supplementary Figure S6.** Time-dependent ROC curves for 1-, 3-, and 5-year OS in the training cohort (A) and validation cohort (B).

**Supplementary Table S1.** Definitions and formulas of blood-based biomarkers

| **Index** | **Full name** | **Formula** |
| --- | --- | --- |
| ALBI | Albumin-Bilirubin score | ALBI = 0.66 × log_10_[bilirubin (μmol/L)] − 0.085 × albumin (g/L) |
| ALBI grade | ALBI grade | Grade 1: ≤ −2.60; Grade 2: > −2.60 to ≤ −1.39; Grade 3: > −1.39 |
| PNI | Prognostic Nutritional Index | PNI = albumin (g/L) + 5 × lymphocyte (×10^9^/L) |
| NLR | Neutrophil-to-Lymphocyte Ratio | NLR = neutrophil (×10^9^/L) / lymphocyte (×10^9^/L) |
| PLR | Platelet-to-Lymphocyte Ratio | PLR = platelet (×10^9^/L) / lymphocyte (×10^9^/L) |
| SII | Systemic Immune-Inflammation Index | SII = [platelet (×10^9^/L) × neutrophil (×10^9^/L)] / lymphocyte (×10^9^/L) |
| HRR | Hemoglobin-to-RDW ratio | HRR = hemoglobin (g/L) / RDW-CV (%) |
| SIRI | Systemic Inflammation Response Index | SIRI = [neutrophil (×10^9^/L) × monocyte (×10^9^/L)] / lymphocyte (×10^9^/L) |
| NAR | Neutrophil-to-Albumin Ratio | NAR = neutrophil (×10^9^/L) / albumin (g/L) |
| HALP | Hemoglobin-Albumin-Lymphocyte-Platelet score | HALP = [hemoglobin (g/L) × albumin (g/L) × lymphocyte (×10^9^/L)] / platelet (×10^9^/L) |
| LMR | Lymphocyte-to-Monocyte Ratio | LMR = lymphocyte (×10^9^/L) / monocyte (×10^9^/L) |

**Supplementary Table S2.** SIS calculation method

| **Variable** | **Definition / Formula** | **Scoring rule** |
| --- | --- | --- |
| Albumin | Serum albumin (g/L) | Alb ≥ 40 → “High Alb”; Alb < 40 → “Low Alb” |
| Lymphocyte-to-monocyte ratio (LMR) | lymphocyte count (×10⁹/L) / monocyte count (×10⁹/L) | LMR ≥ 4.44 → “High LMR”; LMR < 4.44 → “Low LMR” |
| Systemic inflammation score (SIS) | Determined using Alb and LMR | SIS = 0: High Alb and High LMR  SIS = 1: Low Alb or Low LMR  SIS = 2: Low Alb and Low LMR |

**Supplementary Table S3.** Missing Data Summary

| **Variable** | **AFP** | **BMI** | **Operative time** | **Intraoperative blood loss** | **Tumor diameter** |
| --- | --- | --- | --- | --- | --- |
| n (%) | 72(8.6%) | 54 (6.4%) | 24 (2.9%) | 23 (2.7%) | 17 (2.0%) |

**Supplementary Table S4.** VIF for PNII scoring variables

| **Variable** | **PNI** | **ALBI** | **SIS** | **NAR** | **HRR** | **FIB** |
| --- | --- | --- | --- | --- | --- | --- |
| VIF | 4.86 | 4.03 | 2.02 | 1.18 | 1.16 | 1.05 |

**Supplementary Table S5.** Univariable and multivariable Cox regression analyses for OS in the training cohort.

| Variables | Univariable analysis | | Multivariable analysis | |
| --- | --- | --- | --- | --- |
|  | HR (95%CI) | *P* value | HR (95%CI) | *P* value |
| Age (years) |  |  |  |  |
| >66 | Ref. |  | Ref. |  |
| ≤66 | 0.59 (0.44-0.79) | < 0.001 | 0.71 (0.53-0.96) | 0.026 |
| Sex |  |  |  |  |
| Female | Ref. |  |  |  |
| Male | 1.17 (0.85-1.60) | 0.345 |  |  |
| Smoking |  |  |  |  |
| No | Ref. |  |  |  |
| Yes | 1.26 (0.91-1.73) | 0.163 |  |  |
| Drinking |  |  |  |  |
| No | Ref. |  |  |  |
| Yes | 0.89 (0.61-1.29) | 0.545 |  |  |
| BMI (kg/m^2^) |  |  |  |  |
| <18.5 | Ref. |  | Ref. |  |
| ≥18.5 | 0.67 (0.46-0.95) | 0.026 | 0.90 (0.61-1.33) | 0.608 |
| NRS2002 |  |  |  |  |
| ≥3 | Ref. |  | Ref. |  |
| <3 | 0.61 (0.46-0.80) | < 0.001 | 0.92 (0.68-1.25) | 0.608 |
| Tumor location |  |  |  |  |
| Distal | Ref. |  |  |  |
| Proximal | 1.25 (0.95-1.64) | 0.108 |  |  |
| ASA |  |  |  |  |
| >2 | Ref. |  |  |  |
| ≤2 | 0.92 (0.68-1.26) | 0.611 |  |  |
| Operative time (min) | 1.00 (1.00-1.00) | 0.320 |  |  |
| Surgical approach |  |  |  |  |
| Laparoscopic | Ref. |  |  |  |
| Open | 1.09 (0.83-1.44) | 0.543 |  |  |
| Resection extent |  |  |  |  |
| Subtotal | Ref. |  | Ref. |  |
| Total | 2.17 (1.61-2.93) | < 0.001 | 1.31 (0.93-1.86) | 0.123 |
| Reconstruction type |  |  |  |  |
| Billroth I | Ref. |  | Ref. |  |
| Billroth II | 2.57 (0.87-7.59) | 0.088 | 1.99 (0.67-5.98) | 0.218 |
| Roux-en-Y | 3.88 (1.44-10.47) | 0.007 | 2.15 (0.76-6.04) | 0.148 |
| Others | 5.29 (1.82-15.37) | 0.002 | 3.08 (1.00-9.45) | 0.050 |
| Intraoperative blood loss (mL) | 1.00 (1.00-1.00) | < 0.001 | 1.00 (1.00-1.00) | 0.309 |
| Blood transfusion |  |  |  |  |
| No | Ref. |  | Ref. |  |
| Yes | 1.58 (1.19-2.10) | 0.002 | 1.04 (0.76-1.43) | 0.794 |
| Albumin transfusion |  |  |  |  |
| No | Ref. |  |  |  |
| Yes | 1.33 (0.97-1.82) | 0.073 |  |  |
| Diameter (cm) |  |  |  |  |
| >3.5 | Ref. |  | Ref. |  |
| ≤3.5 | 0.33 (0.24-0.46) | < 0.001 | 0.89 (0.62-1.27) | 0.512 |
| Differentiation |  |  |  |  |
| Poorly | Ref. |  | Ref. |  |
| Well to moderately | 0.48 (0.35-0.66) | < 0.001 | 0.72 (0.51-1.01) | 0.059 |
| pT |  |  |  |  |
| T1 | Ref. |  | Ref. |  |
| T2 | 1.65 (0.77-3.58) | 0.201 | 1.05 (0.48-2.32) | 0.902 |
| T3 | 7.22 (3.88-13.41) | < 0.001 | 2.91 (1.41-6.00) | 0.004 |
| T4 | 6.45 (3.56-11.68) | < 0.001 | 2.15 (1.05-4.38) | 0.036 |
| pN |  |  |  |  |
| N0 | Ref. |  | Ref. |  |
| N1 | 2.07 (1.29-3.30) | 0.002 | 1.54 (0.92-2.57) | 0.103 |
| N2 | 4.16 (2.74-6.32) | < 0.001 | 2.24 (1.36-3.70) | 0.002 |
| N3 | 6.22 (4.27-9.07) | < 0.001 | 2.86 (1.74-4.69) | < 0.001 |
| Combined resection |  |  |  |  |
| No | Ref. |  |  |  |
| Yes | 1.19 (0.79-1.80) | 0.405 |  |  |
| Perineural invasion |  |  |  |  |
| No | Ref. |  | Ref. |  |
| Yes | 2.64 (2.00-3.47) | < 0.001 | 1.26 (0.92-1.73) | 0.150 |
| Lymphovascular invasion |  |  |  |  |
| No | Ref. |  | Ref. |  |
| Yes | 2.91 (2.15-3.94) | < 0.001 | 0.93 (0.63-1.39) | 0.723 |
| PNII |  |  |  |  |
| High | Ref. |  | Ref. |  |
| Low | 0.38 (0.28-0.51) | < 0.001 | 0.47 (0.35-0.65) | < 0.001 |
| AFP (ng/mL) |  |  |  |  |
| High | Ref. |  |  |  |
| Low | 0.79 (0.44-1.42) | 0.437 |  |  |
| CEA (ng/mL) |  |  |  |  |
| High | Ref. |  | Ref. |  |
| Low | 0.64 (0.47-0.87) | 0.004 | 1.10 (0.79-1.53) | 0.586 |
| CA125 (U/mL) |  |  |  |  |
| High | Ref. |  | Ref. |  |
| Low | 0.46 (0.29-0.71) | < 0.001 | 0.43 (0.27-0.69) | < 0.001 |
| CA199 (U/mL) |  |  |  |  |
| High | Ref. |  | Ref. |  |
| Low | 0.55 (0.39-0.77) | < 0.001 | 1.10 (0.76-1.60) | 0.600 |

BMI, body mass index; ASA, American Society of Anesthesiologists; PNII, Prognostic Nutritional-Inflammatory-Immune score; OS, overall survival; HR, hazard ratio, CI, confidence interval.

**Supplementary Table S6.** Multicollinearity assessment using generalized variance inflation factor (GVIF)

| **Predictor** | **GVIF** | **Df** | **Adjusted GVIF (GVIF^(1/(2*Df)))** |
| --- | --- | --- | --- |
| Age | 1.06 | 1.00 | 1.03 |
| BMI | 1.16 | 1.00 | 1.08 |
| NRS2002 | 1.23 | 1.00 | 1.11 |
| Resection extent | 1.33 | 1.00 | 1.15 |
| Reconstruction type | 1.46 | 3.00 | 1.06 |
| Intraoperative blood loss | 1.20 | 1.00 | 1.09 |
| Blood transfusion | 1.23 | 1.00 | 1.11 |
| Diameter | 1.32 | 1.00 | 1.15 |
| Differentiation | 1.15 | 1.00 | 1.07 |
| pT | 1.84 | 3.00 | 1.11 |
| pN | 2.00 | 3.00 | 1.12 |
| Perineural invasion | 1.32 | 1.00 | 1.15 |
| Lymphovascular invasion | 1.72 | 1.00 | 1.31 |
| PNII | 1.17 | 1.00 | 1.08 |
| CEA | 1.17 | 1.00 | 1.08 |
| CA125 | 1.13 | 1.00 | 1.06 |
| CA199 | 1.16 | 1.00 | 1.08 |

**Supplementary Table S7.** Event Distribution by CA125 Status

| **Cohort** | **CA125 Status** | **n** | **Deaths, n (%)** |
| --- | --- | --- | --- |
| Training | Normal | 552 | 185 (33.5%) |
| Training | Elevated | 36 | 22 (61.1%) |
| Validation | Normal | 238 | 89 (37.4%) |
| Validation | Elevated | 15 | 3 (20.0%) |
